# Supplementary material for: Determining the rate of infectious disease testing through contagion potential
Source: PLOS Glob Public Health. 2023 Aug 2;3(8):e0002229. doi: 10.1371/journal.pgph.0002229 (PMC10395932; doi:10.1371/journal.pgph.0002229)
Supplement: Attachment — Submitted filename: Rebuttal.pdf [file pgph.0002229.s001.pdf]

Determining the Rate of Infectious Disease Testing through Contagion Potential

PGPH-D-22-01697

Response to comments

February 12, 2023

We thank the editor and reviewer for critically assessing the materials and providing suggestions that significantly improved the presentation of the paper. All editorial changes made during the revision are highlighted in blue color. Below is a summary of changes and the itemized responses to the comments.

Summary of the major changes

1. In Sec. 2.1.3 of the revised manuscript, we have introduced the Human Cell Mobility Model (HCMM) as the means to model superspreader events. We discuss that as per the HCMM model, people tend to visit places that are inhabited by members of their social groups. We elaborate on how defining zones that inhabit a large number of individuals can help model superspreader events, where large social gatherings can be formed.
2. We discuss that another factor influencing the transmissibility and virulence of the virus is its strain. We model the infectivity of different strains by incorporating its basic reproduction number R_0 in the rate parameter β, as $\beta = R_0 \times \gamma$.
3. In Sec. 3.2 of the revised manuscript, we have added a new experiment on the spatial SIRS epidemic model, where we demonstrate that the $\mu N - I$ score is able to capture the true infection trends as compared to the tested infection numbers, under variations of other factors, namely, testing rates, virus strain, reinfection, and superspreader events.
4. We have rewritten parts of the manuscript (and marked the changes in blue) to enhance readability and improve the overall presentation of the manuscript.

Editor's Comment

Overview: The reviewers have recommended further discussion on certain confounding variables, such as the rate of infection and the prevalence of super spreaders and chronically infected individuals, and how they may have affected the results. Their comments can be found below.

Response: In the revised manuscript, we have included a new study on the effects of reinfection, virus strains, and superspreader events on the efficacy of the proposed $\mu N - I$ scores. We have experimentally demonstrated that $\mu N - I$ score captures the true infection numbers more accurately than the tested infected count under all the scenarios.

Reviewer 1

Overview: The authors propose to extend the concept of Contagion Potential (CP) to assess the dynamics of COVID-19 transmission, using spatial and SIR models. The concepts, assumptions, and equations are coherent, and the inclusion of mobility measures, as well as vaccination and testing, as factors influencing the dynamics of SARS-Cov-2 transmission is appropriate. Results are compelling, especially for contributing to public policies in a controversial topic, i.e., the importance of lockdown or other nonpharmaceutical measures.

Response: We thank the reviewer for acknowledging the merit of this work. Indeed, we are hopeful that the proposed metric, contagion potential (CP), will capture the true dynamics of COVID-19 transmission and inform public policies on pandemic management. As per the recommendation, in course of revision, we have included a new study on the efficacy of the proposed metric, $\mu N - I$ in capturing the true infection numbers under variations in different factors, namely, testing rates, reinfection, superspreader events, and virus strains.

In course of the revision, we have introduced the Human Cell Mobility Model (HCMM) [1, 2] as the means to model superspreader events. We discuss that as per the HCMM model, people tend to visit places that are inhabited by members of their social groups. We elaborate on how defining zones that inhabit a large number of individuals can help model superspreader events, where large social gatherings can be formed (refer to Sec. 2.1.3 of the revised manuscript).

We study that another factor influencing the transmissibility of the virus is its strain. We model the infectivity of different strains by incorporating its basic reproduction number R_0 in the rate parameter β, as $\beta = R_0 \times \gamma$ [3]. In Sec. 3.2 of the revised manuscript, we have added a new experiment on the spatial SIRS epidemic model, where we demonstrate that the $\mu N - I$ score is able to capture the true infection trends as compared to the tested infection numbers, under variations of other factors, namely, testing rates, virus strain, reinfection, and superspreader events.

References

[1] C. Boldrini and A. Passarella. Hcmm: Modelling spatial and temporal properties of human mobility driven by users' social relationships. *Computer Communications*, 33(9):1056–1074, 2010.

[2] G. Solmaz and D. Turgut. A survey of human mobility models. *IEEE Access*, 7:125711–125731, 2019.

[3] I. Korolev. Identification and estimation of the seird epidemic model for covid-19. *Journal of econometrics*, 220(1):63–85, 2021.
