# Supplementary material for: Determining the rate of infectious disease testing through contagion potential
Source: PLOS Glob Public Health. 2023 Aug 2;3(8):e0002229. doi: 10.1371/journal.pgph.0002229 (PMC10395932; doi:10.1371/journal.pgph.0002229)
Supplement: Attachment — Submitted filename: Response to reviewers.pdf [file pgph.0002229.s002.pdf]

# Determining the Rate of Infectious Disease Testing through Contagion Potential

PGPH-D-22-01697R1

Response to comments

June 7, 2023

We thank the editor and reviewer for critically assessing the materials and providing suggestions that significantly improved the presentation of the paper. All editorial changes made during the revision are highlighted in blue color. Below is a summary of changes and the itemized responses to the comments.

## Summary of the major changes

1. We have rewritten parts of the introduction section to discuss existing literature on the need to incorporate spatiotemporal context into epidemiological studies in order to improve spread predictions. These works touch upon the modifications of the epidemic model to create age- and gender-based compartmentalization as well as the use of time-varying model parameters.

2. We have added a new section (refer to Sec. 2.3) on the optimization of profit and turnover in an industrial setting to demonstrate how contagion potential (CP) captures the effect of infection spread and vaccination policies of industries on their productivity and profit margins. Using a realistic human mobility dataset, we experimentally show that CP captures the effect of vaccination policies of industries on their contagion rates, worker output, and the consequent industrial revenue trends over time (sec Sec. 3.5).

3. We have rewritten parts of the discussion section to elucidate how the present work differs from existing efforts as well as the efficacy of CP as a metric to analyze infectivity at a population and individual level. Furthermore, we discuss the limitations of the proposed metric as well as our current research efforts to address them.

4. We have rewritten other parts of the abstract and main text in the revised manuscript to eliminate typographic errors and enhance readability.

## Reviewer 3

**Comment:** Overall, the study is interesting but I have several issues in its current form:

**Response:** We thank the reviewer for the comments. We have incorporated the queries in the course of revision.

**Comment:** The authors are focusing on a very complex and well-studied subject while not providing a separate Related Work section. I do not say they have to, and can put it as part of the Introduction section but must provide the user with a more detailed review of the subject.

**Response:** We appreciate the reviewer's suggestion. In Sec. 1 of the revised manuscript, we have discussed the references suggested by the reviewer (and a few others) [1, 2, 3, 4, 5, 6, 7, 8]. These cover the efforts to incorporate spatiotemporal context into epidemiological studies and improve spread predictions. Specifically, they include

- the adaptation of the existing epidemic model to create age- and gender-based compartmentalization and proposing enforcing distancing measures based on ICU occupancy or combining it with spatial diffusion models to predict contagion;
- time-varying model parameters and context information such as population flow, meteorological factors, etc.

**Comment:** The mobility and social mixing approach is interesting but should be better supported with real data. For example, `https://www.sciencedirect.com/science/article/pii/S0038012123000538` used a graph-based epidemiological model where individuals move between nodes according to logic and the nodes are different. They support it with historical records. I suggest the authors would do the same.

**Response:** We have incorporated the reviewer's suggestion by adding a new section on industrial optimization. Specifically, we adapt the optimization formulation (in Sec. 2.3 of the revised manuscript) proposed in the stated paper into the system model of the present work. The goal is to demonstrate how contagion potential (CP) captures the effect of infection spread and vaccination policies of industries on their productivity and profit margins. To model realistic human mobility, we leverage a dataset taken from the mobility-flow dataset in the US during COVID-19 [9] to generate human zone transition probability. The experimental results discussed in Sec. 3.5 shows that CP captures the effect of vaccination policies of industries on their contagion rates, worker output, and the consequent industrial revenue trends over time.

**Comment:** The model's limitations and applications should be discussed better.

**Response:** We have rewritten parts of the discussion section (Sec. 4) of the revised manuscript. We discussed that, unlike existing efforts that adapt SIR and SEIR epidemic models to integrate spatial, socio-demographic, clinical, and temporal contexts, the proposed contagion potential (CP) metric measures infectivity at a population-level and individual level and it leverages real epidemiological data and epidemic models as inputs of daily infected, recovered statistics, epidemic models, etc. Furthermore, we briefly cover the following future research directions, on the

- creation of variants of CP that will dynamically track the phase shifts in $\mu N - I$ and inform public policies even from partial population knowledge.
- inclusion of CP into contact-tracing applications, which would gauge the relative risk of a person in embarking on a trip and schedule their mobility to minimize the risk of contagion.

**Comment:** How are the values in Table 3 obtained?

**Response:** We have added a discussion and references at the beginning of Sec. 3 of the revised manuscript to explain how the recovery and reinfection parameters $(\gamma, \delta)$ are determined. Furthermore, we explain that the infectivity rate has been estimated by plugging in the viral load shedding period cited in the literature [10] of $K = 21$ days into Eq. 10 of the manuscript.

**Comment:** Explain the bars in Fig. 4 - I guess they are one STD but this is not clear from the caption.

**Response:** We rewrote the caption of Fig. 4. We stated that the figure shows the effect of population density, spread parameter $\beta$, and velocity on the average contact rate and infected numbers. We also pointed out that the error bars signify a standard deviation from the mean, as follows:

*Infectivity in a Spatial Model: (a) average contact rate among individuals for varying population density in a spatial model; (b) the number of newly infected for varying rates of disease spread $\beta$ under the assumption of (b) binary infectivity and (c) continuous infectivity; and (d) increase in the average number of contacts with the increase in individual velocity. The error bars signify a standard deviation from the mean.*

**Comment:** The design of Fig 6 color-wise is strange.

**Response:** We have redrawn the figure to improve the alignment of the subfigures at the top and bottom of Fig. 6.

# References


[1] V. Ram and L. Schaposnik. A modified age-structured sir model for covid-19 type viruses. *Scientific reports*, 11(1):15194, 2021.

[2] G. Kozyreff. Hospitalization dynamics during the first covid-19 pandemic wave: Sir modelling compared to belgium, france, italy, switzerland and new york city data. *Infectious disease modelling*, 6:398–404, 2021.

[3] A. Viguerie et al. Simulating the spread of covid-19 via a spatially-resolved susceptible–exposed–infected–recovered–deceased (seird) model with heterogeneous diffusion. *Applied Mathematics Letters*, 111:106617, 2021.

[4] T. Lazebnik and A. Alexi. Comparison of pandemic intervention policies in several building types using heterogeneous population model. *Communications in Nonlinear Science and Numerical Simulation*, 107:106176, 2022.

[5] Z. Wang, X. Zhang, G. Teichert, M. Carrasco-Teja, and K. Garikipati. System inference for the spatio-temporal evolution of infectious diseases: Michigan in the time of covid-19. *Computational Mechanics*, 66:1153–1176, 2020.

[6] P. Holme. Fast and principled simulations of the sir model on temporal networks. *Plos one*, 16(2):e0246961, 2021.

[7] Y. Chen, Q. Li, H. Karimian, X. Chen, and X. Li. Spatio-temporal distribution characteristics and influencing factors of covid-19 in china. *Scientific Reports*, 11(1):1–12, 2021.

[8] T. Lazebnik and G. Blumrosen. Advanced multi-mutation with intervention policies pandemic model. *IEEE Access*, 10:22769–22781, 2022.

[9] Y. Kang, S. Gao, Y. Liang, M. Li, and J. Kruse. Multiscale dynamic human mobility flow dataset in the u.s. during the covid-19 epidemic. *Scientific Data*, pages 1–13, 2020.

[10] S. Malhotra et al. Sars-cov-2 reinfection rate and estimated effectiveness of the inactivated whole virion vaccine bbv152 against reinfection among health care workers in new delhi, india. *JAMA network open*, 5(1):e2142210–e2142210, 2022.
