# Supplementary material for: Determining the rate of infectious disease testing through contagion potential
Source: PLOS Glob Public Health. 2023 Aug 2;3(8):e0002229. doi: 10.1371/journal.pgph.0002229 (PMC10395932; doi:10.1371/journal.pgph.0002229)
Supplement: Attachment — Submitted filename: Rebuttal.pdf [file pgph.0002229.s003.pdf]

Determining the Rate of Infectious Disease Testing through Contagion Potential

PGPH-D-22-01697R2

Response to comments

June 29, 2023

We thank the editor and reviewer for critically assessing the materials and providing suggestions that significantly improved the presentation of the paper. All editorial changes made during the revision are highlighted in blue color. Below is a summary of changes and the itemized responses to the comments.

Summary of the changes

1. In the introductory section, we included the reference suggested by the editor.
2. We regenerated Fig. 7 and rewrote its description (in Sec. 3.2) to demonstrate that contagion potential (CP) captures the change in infectivity due to varying reproduction numbers.
3. We rewrote the entire discussion section (Sec. 4) to elucidate how this relates to any other previous work and its implications in future pandemic management policymaking.
4. We have rewritten other parts of the abstract and main text in the revised manuscript to eliminate typographic and reference number errors and enhance readability.

Editor's Comment

Comment: Several peer reviews have already reviewed the paper and I believe the authors have responded well to their comments on the methods and results. I do think the discussion needs to be fleshed out a bit more. I would recommend having a (brief) paragraph discussing how this relates to any other previous work. It also could be helpful to explain out a bit more what the implications of the contagion potential are, especially to a more general audience who may not be as familiar with mathematical modeling.

Response: We have rewritten the discussion section (Sec. 4), explaining that our approach explores a metric known as contagion potential (CP), which serves as a measure of the infectivity profile within a specific zone. It is important to note that CP does not modify existing epidemic

models. Instead, it provides a complementary analysis by quantifying the infectivity of individuals and aggregated to learn the infectivity profile of a geographical region. This unique metric can be applied to any temporal infection data of which the epidemic model can be a potential source. By leveraging CP, we gain valuable insights into the level of infectivity present within a zone, allowing us to better understand the transmission dynamics of infectious diseases. Furthermore, we demonstrate that CP can be effectively estimated using various sources of data, such as social contact information at an individual level (such as contact data) or temporal epidemiological data at a population level, which includes daily infected and recovered statistics. By incorporating CP into our analysis, we enhance our ability to assess the impact of infections and develop more effective strategies to mitigate their spread. The integration of contagion potential (CP) into public policy may change decision-making processes and shape the future of infectious disease control. By leveraging CP as a measure of the infectivity profile within specific geographical zones, policymakers can gain insights into

- targeted and evidence-based intervention strategies,
- the infectivity levels present in different areas, allowing for a more nuanced approach to public health efforts,
- knowledge of high-risk zones and allocate resources, accordingly, focusing on areas with elevated CP to prioritize testing, contact tracing, and targeted interventions,
- effectiveness of earlier measures based on evolving CP, allowing for timely adjustments to control strategies, and
- optimization of resource allocation, minimization of the impact on healthcare systems, and enhancement of overall disease management.

CP can extend beyond infectious diseases, as similar approaches may be applied to other areas such as public safety, disaster response, and environmental health, providing a versatile tool for evidence-based decision-making across domains. As we move into the future, the integration of CP into public policies holds great promise in enabling proactive and effective responses to emerging infectious threats, ultimately safeguarding public health and well-being on a global scale.

Comment: Is there a way to distinguish it colloquially from R_0? Also, could contagion potential be used to explain epidemic potential at different geographical levels? (e.g., Masters et al. `http://www.pnas.org/lookup/doi/10.1073/pnas.2011529117`)

Response: We regenerated Fig. 7 and added a discussion in Sec. 3.2 to show that contagion potential (CP) μ captures the change in infectivity due to varying reproduction numbers (R_0). Specifically, we compare strains with different R_0 keeping other factors (i.e., mobility and reinfection rate) unchanged. The time-evolving value of μ for Omicron (refer to Fig. 7(c) of the revised manuscript) is shown to be higher than that of Delta (Fig. 7(a)), accounting for increased infectivity from Omicron ($R_0 = 9.5$) than Delta ($R_0 = 3.2$) Also, we have incorporated the mentioned paper (`http://www.pnas.org/lookup/doi/10.1073/pnas.2011529117)`) as part of literature review in the introductory section of the revised manuscript.

Finally, as per the editor's recommendation, we explain in the discussion section that while the reproduction number (R_0) is a well-established measure of infectivity, the CP metric introduces a distinct and complementary perspective in understanding the dynamics of infectious diseases. R_0

primarily focuses on the intrinsic characteristics of a pathogen, such as its virulence and transmissibility, whereas CP considers the additional influence of social contact resulting from mobility patterns. It recognizes that the spread of diseases is not solely determined by the inherent properties of the pathogen, but also by human behavior, contact, and interactions. By incorporating social contact data and considering the movement of individuals within geographical locations, CP captures the effect of mobility and the resultant increased scope for transmission. This aspect sets CP apart from R_0, as it provides a more comprehensive assessment of infectivity by accounting for the interplay between pathogen characteristics and human behavioral factors. As discussed above, we experimentally show in Sec. 3.2 that CP captures the independent effect of varying R_0 due to different viral strains on the infectivity profile. Going forward, a combination of such sociodemographic, mobility, and epidemiological factors may enable policymakers and researchers to gain insights into the mechanisms driving disease transmission and devise targeted interventions and public health strategies to address the interconnectedness of human interactions and infectious disease dynamics. The goal of our ongoing and future studies will be to

- create simulation and numeric experimental designs to compare how the CP profiles change with differing R_0 profiles,
- validate the efficacy of this model using real data and agent-based simulations, and
- incorporate CP into contact-tracing applications to gauge the relative risk of a person in embarking on a trip and schedule their mobility to minimize the risk of contagion.

Reviewer 3

Comment: All comments have been addressed.

Response: We thank the reviewer for his constructive feedback.
